# Do graspable objects always leave a motor signature? A study on memory traces

**DOI:** 10.1007/s00221-022-06487-4

**Published:** 2022-10-22

**Authors:** Elena Daprati, Priscilla Balestrucci, Daniele Nico

**Affiliations:** 1 Dipartimento di Medicina dei Sistemi, Università di Roma Tor Vergata, Via Montpellier 1, 00133 Rome, Italy; 2 Faculty for Computer Science, Engineering, and Psychology, Applied Cognitive Psychology, Ulm University, 89081 Ulm, Germany; 3 Dipartimento di Psicologia, Università di Roma la Sapienza, 00185 Rome, Italy

**Keywords:** Concrete concepts, Picture superiority effect, Dynamic superiority effect, Enactment effect, Motor cognition, First-person experience

## Abstract

Several studies have reported the existence of reciprocal interactions between the type of motor activity physically performed on objects and the conceptual knowledge that is retained of them. Whether *covert* motor activity plays a similar effect is less clear. Certainly, objects are strong triggers for actions, and motor components can make the associated concepts more memorable. However, addition of an action-related memory trace may not always be automatic and could rather depend on ‘how’ objects are encountered. To test this hypothesis, we compared memory for objects that passive observers experienced as verbal labels (the word describing them), visual images (color photographs) and actions (pantomimes of object use). We predicted that the more direct the involvement of action-related representations the more effective would be the addition of a motor code to the experience and the more accurate would be the recall. Results showed that memory for objects presented as words i.e., a format that might only indirectly prime the sensorimotor system, was generally less accurate compared to memory for objects presented as photographs or pantomimes, which are more likely to directly elicit motor simulation processes. In addition, free recall of objects experienced as pantomimes was more accurate when these items afforded actions performed towards one’s body than actions directed away from the body. We propose that covert motor activity can contribute to objects’ memory, but the beneficial addition of a motor code to the experience is not necessarily automatic. An advantage is more likely to emerge when the observer is induced to take a first-person stance during the encoding phase, as may happen for objects affording actions directed towards the body, which obviously carry more relevance for the actor.

## Introduction

Ogni oggetto [è] comportamento trasformato in cosa, e poi ritrasformato in comportamento—Daniele Del Giudice, Atlante occidentale (1985).^1^

When asked to provide a definition of a manipulable item, such as a pen or a hammer, most people supply a functional, action-related, description: a pen is an instrument for writing, a hammer is a tool used to pound nails. These functional definitions are among the earliest descriptions to emerge during language development. In a definition task, 5-year-old children mainly provided functional or physical descriptions of object-words (e.g., “a ladder is to reach high”) (McGregor et al. 2002) and a similar attitude was reported in blind children (Anderson 1984; Vinter et al. 2013) supporting the idea that the “meaning of an object […] is a pattern of possible actions” (Glenberg 1997, p.4). These observations are not surprising: our experience of the world is mediated by the sensory and motor systems. Information conveyed by vision, smell or touch contribute to create rich multimodal descriptions of our surroundings and the motor system provides the means to navigate the environment and interact with its elements, further characterizing physical objects. Accordingly, it seems logical to assume that information derived from sensorimotor experiences contribute to object representations and can be easily retrieved and used when planning novel interactions or remembering past ones.

In line with this view, activity in cortical areas engaged when objects are actively used has been systematically described also during retrieval and/or manipulation of information about object properties (Martin 2007, 2016). Likewise, the neural circuits involved in transforming object affordances into hand actions were activated when information about graspable objects was maintained in working memory (Mecklinger et al. 2002). In fact, activity in motor areas seems to be engaged also when viewing object nouns (Horoufchin et al. 2018), reasoning on them (Marino et al. 2013) or simply thinking about manipulable objects (Yee et al. 2013), suggesting that dealing with object concepts systematically recruits the motor system even when no overt movement is required and/or objects are not physically present. Interestingly, albeit *covert*, this motor involvement is powerful enough to interfere with on-going overt actions: if participants automatically read (Gentilucci et al. 2000; Gentilucci 2003) or listen to (Boulenger et al. 2006; Nazir et al. 2008) words describing object properties when reaching for a target, the kinematics of their arm movements undergoes distinctive modifications.

In the same way as object concepts appear to recruit the sensorimotor system, overt actions have been reported to affect verbal tasks involving manipulable objects. In categorization tasks requiring to decide whether an object is natural or manufactured, the type of action performed to respond influenced response latencies, and this was proved true also if the objects appeared as black and white photographs or object names (Tucker and Ellis 2004). A similar phenomenon was found when participants were called to respond with an action toward or away from their body when deciding if a sentence made sense or not: congruency effects emerged when the action described by the sentence and that used for the response matched (Action-sentence Compatibility Effect, ACE; Glenberg and Kaschak 2002). Priming effects were also reported whereby the action afforded by a named object speeded verbal responses to the next item: a plier for example, was named faster if preceded by a nutcracker compared to when it was preceded by an object affording an entirely different movement (Helbig et al. 2006, 2010; Myung et al. 2006).

Taken together, these data clearly underline a close relationship between objects and actions. Whether this link relies on the capacity of objects to systematically elicit the actions they afford, or on motor information partaking of objects’ conceptual representations is less clear. In categorization tasks like the one mentioned above, Tucker and Ellis (2001) reported comparable effects when stimuli appeared in the near (15 cm) and far space (2 m), i.e., independently from whether a direct interaction with the object was physically possible. Although this observation suggests that some motor properties could be integral to object representations, more recent findings rather describe a context-dependent effect of object affordances (Costantini et al. 2010, 2011; Ranganathan et al. 2011; Borghi and Riggio 2009, 2015; Wokke et al. 2016; Orban et al. 2021a). For example, in a task requiring healthy participants to reproduce a seen grip, a compatibility effect emerged between the moving hand and the handle of the object used as go-signal, *but* only if the object was presented within the reaching space. No such effect was found if the object was out of reach, either because located at 1.5 m from the participant (Costantini et al. 2010; Exp1) or behind a semi-opaque screen (ibid., Exp2). Distance-related effects were also reported in a behavioral study using object pictures as primes for action verbs (Costantini et al. 2011), and in animal experiments recording object-triggered responses from premotor neurons, which showed similar space-constrained modulation (Bonini et al. 2014; Maranesi et al. 2014) as well as different dynamics based on the context in which objects are presented (Maranesi et al. 2019). A possible account for these contrasting findings has been proposed (Borghi and Riggio 2009, 2015) whereby a distinction is made between stable and variable affordances. While stable affordances would depend on invariant object properties (e.g., cherries are always grasped with a precision grip, while apples typically require a power grip), variable affordances would be directly linked to the action one is about to perform and would thus be influenced by ‘temporary’ objects features, such as their location (e.g., whether a cherry is on a tree or on a table). This distinction has interesting implications because it suggests that different action-related information could be involved according to how objects are encountered—and may contribute to characterizing the experience of the interaction.

In daily life, objects are indeed encountered in a variety of situations. One can come across a ball when watching a child playing with it or when seeing a ball in a meadow, but one can also encounter a ball when looking at a billboard or reading the word ‘ball’ in a book. Each of these interactions may elicit a covert action involving the object but resonance within the motor system need not be identical. Seeing a ball in a meadow might trigger a kicking or grasping movement (Murata et al. 1997; Grafton et al. 1997) that one may or may not wish to overtly perform. Watching a child bouncing a ball could prompt automatic imitation (Heyes 2011) or invite the observer to initiate an interaction. In fact, observing manipulative actions can elicit an ample variety of behavioral responses based on the context in which the action takes place (cf. social affordance; Orban et al. 2021b). Interestingly, neural activity related to observation of static tools and of the actions involving them follows a precise—and temporally distinct dynamics (Caruana et al. 2017), suggesting that although the sight of objects and actions both affect the sensorimotor system, they may do so at entirely different levels. As for words, when recording from single neurons in high-level sensorimotor cortex of individuals involved in a brain-machine interface study, Aflalo et al. (2020) showed similarities in the responses to real actions and action verbs (such as grasp or push), indicating that words are indeed linked to their sensorimotor representations. On the other hand, it is also true that reading the name of an objects or watching its photo may prime a variety of non-motor information prior—or in addition to, activating the sensorimotor system in response to the object’s affordances (Mahon and Caramazza 2008; Meteyard et al. 2012). Accordingly, it can be assumed that all these action-related phenomena—albeit convergent on the same concept—relate to entirely different experiences and may be stored differently for the purpose of future actions, communications, or thoughts.

In the present study, we were especially interested in the frequent, casual encounters that individuals have with objects, and for which no immediate interaction is intended, required or simply feasible. This happens for example, when one watches commercials on television and is presented with dynamic and static images of objects as well as with a plethora of object names, or when casually looking at billboards along the road while driving. Do these items equally succeed in adding an action-related trace that later helps characterizing the modality of the experience? To answer this question, we compared memory for objects that had been previously presented as action representations with memory for objects that were experienced via visual or verbal representations, for which involvement of the sensorimotor system may represent a more indirect process (Mahon and Caramazza 2008). Specifically, to describe objects in terms of afforded actions, we used videos displaying pantomimes of object use drawn from a pool collected in a previous study and rated as highly recognizable (Daprati et al. 2005). Pantomimes were preferred to interactions with real objects to limit confounds due to the simultaneous observation of both object *and* action, which could have biased the participants’ attention towards the former rather than the latter—which we were specifically interested in testing. Several data from the literature support the notion that pantomimes convey a significant amount of information about the represented object: for example, some kinematic traits relative to objects’ weight are similar in real and pantomimed grasping (Ansuini et al. 2016) and observers can accurately estimate weight from pantomimed actions directed towards invisible objects (Podda et al. 2017), suggesting that this type of stimulus could reliably describe the involved object. We then selected to use color, bidimensional images of objects instead of black-and-white sketches or 3D images to better mimic the common experience of viewing photographs on billboards or television screens. For similar reasons, real objects were excluded as their presentation would have implied interrupting the continuous flow of presentation we wished to obtain on the distal screen. Thus, in an ecological setting that mimicked casual observation of billboards, pictures and movies, healthy participants watched a series of slides projected on a distant wall screen. In random order, each slide could show a word describing an object (WD), a color photograph of an object (PG) or an empty-handed actor pantomiming use of an object (PM). All objects afforded at least one paradigmatic action and the movement performed by the actor clearly referred to the object’s most characteristic use (e.g., paintbrush = painting). No instructions were given except that of watching and no overt movements were required on the part of the participants, who were expected to act merely as passive observers. In separate groups of participants, memory for the presented items was tested by means of either a Free Recall Task or a Recognition Task. In both cases participants were specifically asked about the format in which the objects had been encountered, i.e., as WDs, PGs or PMs (source memory). We reasoned that—although all three depictions may prompt a motoric representation of the presented objects—the more direct the access to action-related representations the more effective would be the resonance within the motor system and the more likely the addition of a motor code to the corresponding memory trace. Hence, we anticipated that participants would show better memory for objects presented as pantomimes and photographs compared to verbal labels. On the other hand, if strength of these memories were more influenced by the match between encoding and retrieval format (Tulving and Thomson 1973), we expected memory for items presented as words to be most accurate because these items were the only ones benefiting from format correspondence, specifically in the Recognition Task.

## Methods

### Participants

The present study was carried out according to the Declaration of Helsinki and was approved by the local ethical committee (Comitato Etico Indipendente Fondazione PTV Policlinico Tor Vergata, Prot. 99/13). All participants provided informed consent before entering the experimental session. Sample size was estimated using G-Power (Faul et al. 2007) assuming alpha = 0.05 and power = 0.80.

Participants were 106 neurologically intact volunteers recruited among students attending local universities. All were native Italian speakers, had normal or corrected-to-normal vision and were naive as to the purpose of the study. Exclusion criteria were history of neurological and/or psychiatric disease and pharmacological treatment involving medicaments interfering with alertness or vigilance (e.g., antihistamines). To check for these parameters, participants completed a form where a list of conditions was presented. Only 2 participants were excluded after this screening.

Sixty-eight participants (*M* = 22.2 SD = 3.1, 39 females, 29 males, 2 left-handers) were assigned to the Free Recall Task and 36 participants (*M* = 22.0, SD = 2.1; 17 females, 19 males; 3 left-handers) to the Recognition Task. The choice to include a larger number of participants in the Free Recall group was because false memories (i.e., novel objects reported as old) are uncommon in healthy individuals in this type of task (Laws and Bhatt 2005). Yet, false memories could be relevant here as they may inform on motor similarity effects comparable to those reported in previous studies (e.g., Helbig et al. 2006, 2010; Myung et al. 2006).

### Stimuli

Stimuli were 36 objects of common use (see Appendix1 for details). For each object there were three possible presentation forms: (i) the Italian word describing the object (WD), written in Arial lowercase black letters on a white background; (ii) a colored photograph of the object (PG) presented on a white background; iii) a pantomime (PM) in which an actor appeared to be using the object. In the latter set, objects were not overtly presented but their identity was clearly described by the actor’s movements. The action performed with the objects was always an extremely paradigmatic and recognizable one, i.e., painting (paintbrush), putting on (glasses, hat), throwing (stones) and the like. The actor was a male, right-handed young man dressed in black clothes, filmed using a medium close shot (head to hip level) against a dark background (for details on the pantomimes, see Daprati et al. 2005).

### Procedure

The experimental session was run in a large lecture room equipped with two wall screens (250 × 190 cm). Participants were tested in small groups of 6–12 individuals each. They comfortably sat in the front rows of a series of writing-pad equipped chairs located at an average distance of 3.5 m from the screens. Each session was organized in two parts and lasted approximately 30 min.

#### Part 1

Participants received a notebook that included the informed consent sheet, a series of interview questions (relative to age, gender, handedness, compliance with inclusion criteria), and a response form showing a list of empty lines. Participants were instructed to read and sign the informed consent, hand it to one experimenter and next complete the interview form. They were also told to keep the response form for later use. Once all participants had completed this preliminary phase, one experimenter read aloud the following instructions: “*In the next 5 min you will see a series of video-clips, words, photos and line drawings. They will be presented on the screen in random order and in a continuous and rather fast sequence so try to keep your gaze on the screen, without getting distracted. At a given point you will be asked to answer some questions. Please do it as quickly as possible so that you don’t miss anything that might be happening on the screen. Unless other instructions appear on the screen, your primary task is to watch what we are showing you, because later you may be interviewed on it*.” At this point, lights were dimmed, and the stimuli were presented, one at a time, in a semi-random sequence (repetition of the same format < 3). Each stimulus was displayed centrally and remained visible for 4 s with an inter-stimulus transition of 0.5 s. Transition was obtained by scrambling one image prior to presenting the next. After the 36 stimuli had been presented (approximately 3 min), instructions appeared on the screen telling participants that they were to complete a decision task about line drawings of geometric shapes. Twelve consecutive items were then presented on the screen, each showing a large polygonal shape on the left side of the slide and a segment on the right. Participants had to decide whether the segment belonged in the shape or not. Each item remained visible on the screen for 8 s and participants responded by writing yes or no on the provided response form. This task concluded part1 and one experimenter collected the response forms.

#### Part 2

A second form was placed (printed-face down) on the participants’ desks. For the Free Recall group, the printed form was divided in four rectangles labeled ‘word’, ‘photo’, ‘pantomime’, and ‘notes’. Position of the labels varied across participants. For the Recognition group, the response sheet was a grid presenting four columns (labeled as ‘word’, ‘photo’, ‘pantomime’, ‘new’) and a space for notes. Position of the labels also varied across participants. Instructions were read aloud by one experimenter and recited as follows: “*In the presentation you have just viewed there were many objects: some of them were presented as photographs, other were described as word labels, other were not physically present but were used in the pantomimes. After the go signal, please turn the sheet you have just received”.* For the Free Recall group instructions continued as follows: “*You will see that there are four boxes labeled word, photo, pantomime and notes. Your task is to write down as many of the objects as you can remember from the previous presentation. If you remember how they were presented write their name in corresponding box otherwise write the name in the space for notes”*. For the Recognition group the text was the following: *“You will see a grid with four columns labeled word, photograph, pantomime, new – not necessarily in this order, and a list of objects’ names. For each object in the list, you must decide whether – in the previous presentation – it appeared as a photograph, as one of the written words, or the actor pretended to use it in the pantomimes. Write an X in the column indicated by the label you believe is correct. If you believe that the object had not been presented before, write an X in the ‘new’ column. If you remember an object but cannot quite place it, write its name in the space for the notes”.* For both groups, the instructions concluded as follows: “*You have 10 min to complete this part, but you will probably be finished long before the time elapses.*” Participants were then given the go-signal and once completion time was over, one experimenter collected the forms. An informal debriefing session followed, in which one experimenter briefly explained the aim of the study and answered participants’ questions.

### Data analyses

For both groups of participants, the proportion of correct answers out of the total number of presented items (*hit rate*, HIT) was separately computed for objects presented as WDs, PGs and PMs.

For the Free Recall task, an item was scored as a ‘hit’ if the object reported had been part of the list of presented items. For objects that could be called with different names, synonyms were accepted if the alternative word was of common use and allowed no ambiguity as to the described object (e.g., in Italian, ‘temperamatite’, ‘temperamatita’, ‘temperino’ are all acceptable names for ‘pencil sharpener’). Responses were further checked for *inversions* (i.e., items presented in one format that were reported as if they were presented in another format) and for *false memories* (i.e., objects that had not been presented but were incorrectly reported as words, photographs, or pantomimes).

For the Recognition task, an item was scored as a ‘hit’ if the participant recognized it was an old item and correctly indicated the format in which it had been presented. In addition, *correct rejections* (CR) were computed, as the proportion of new items correctly described as ‘new’ out of the total number of new items presented in the grid.

All proportions were arcsine transformed before being entered into statistical analyses. Since data distribution did not significantly differ from normal distribution, parametric analyses were run (Shapiro–Wilk test, Free Recall group: 0.97, *p* = 0.16; Recognition group: 0.96, *p* = 0.25). Separate one-way ANOVA were used to explore the effects of presentation format (WD, PG, PM) in the two groups of participants. T-tests were used for post-hoc comparisons and for testing differences relative to action type (see Results).

For all statistics, the alpha level for acceptance was set at 0.05. Bonferroni correction was applied to multiple comparisons whenever appropriate. Statistical analyses were carried out using JASP (JASP Team 2022) and MATLAB software.

## Results

### Free recall

On average, participants correctly recalled 12 of the 36 presented objects (*M* = 12.40, SD = 3.66). The relatively low accuracy score (20–40%) is in line with previous findings (Snow et al. 2014; Dutriaux and Gyselinck 2016; Dutriaux et al. 2019). In total, there were only 51 false memories and 23 inversions (out a total of 917 responses provided, i.e., 5.6% and 2.5%, respectively). In fact, over 90% of the items that participants reported on the form corresponded to objects that had been previously presented (*M* = 0.92, SD = 0.08). Cases in which participants commented ‘not sure’ or ‘don’t know’ were extremely rare (0.2%).

**Fig. 1:**
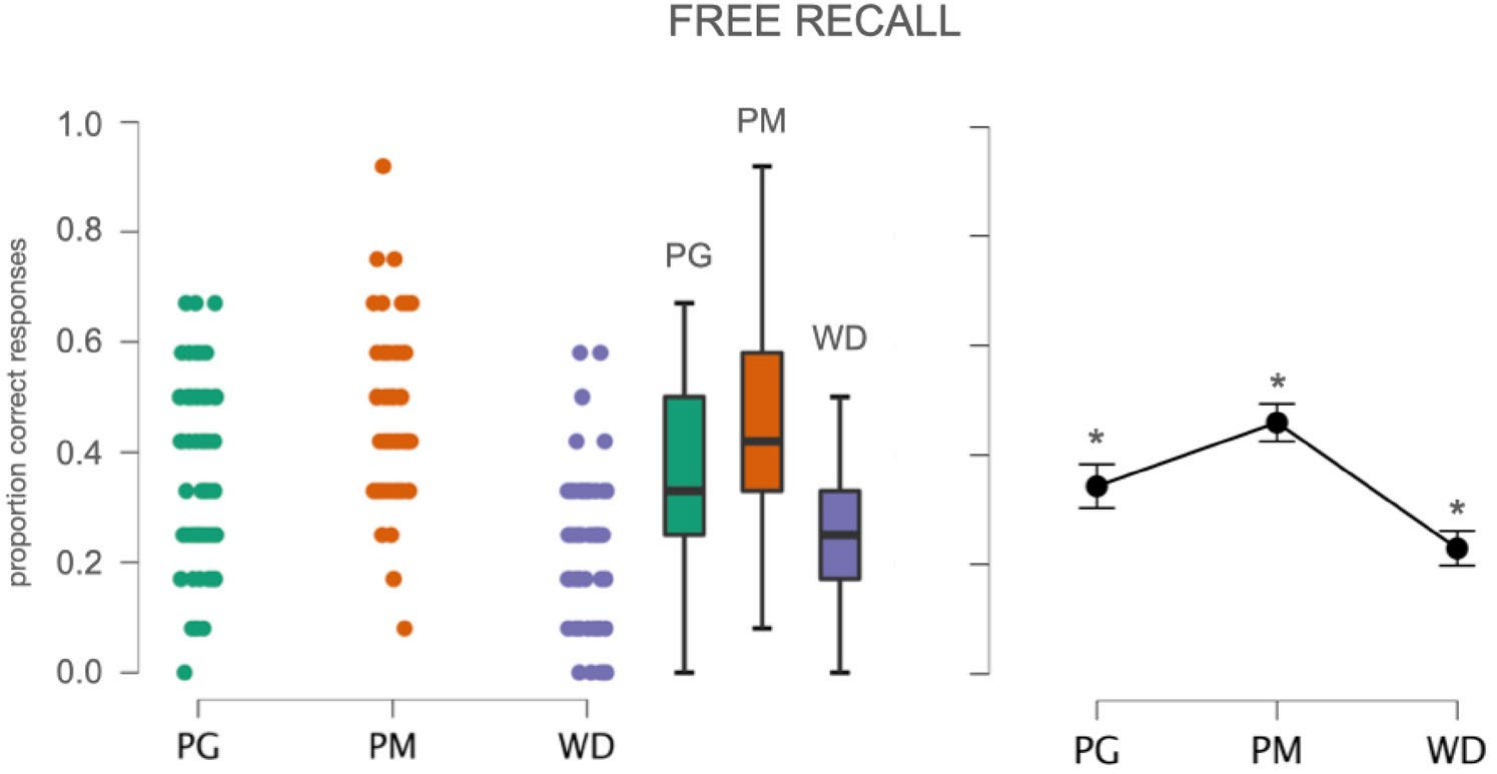
Accuracy for participants in the Free Recall group (*N* = 68). Left: Jitter plots reporting all data points. Centre: Boxplot reporting median values (horizontal lines), interquartile range (top and bottom edges of the boxes). Whiskers extend to the most extreme data points. Right: Mean values (dot marker) and SEM. (whiskers). Asterisks highlight the significant post-hoc comparisons: PG vs. PM, *t* = − 4.26, *p* < 0.0001; PG vs. WD, *t* = 4.51, *p* < 0.00003; PM vs. WD, *t* = 8.77, *p* < 0.000001. *PG* objects presented as photographs, *PM* objects presented as pantomimes, *WD* objects presented as verbal labels

Hit rate, expressed as the proportion of correct answers out of the total number of presented items, was typically higher for objects previously presented as PMs (*M* = 0.46, SD = 0.14) and PGs (*M* = 0.34, SD = 0.17) than for objects that had appeared as WDs (*M* = 0.23, SD = 0.13). A one-way ANOVA on hit rates with presentation format (PG, PM, WD) as within-subjects factor showed a significant main effect of the former, *F*(2,201) = 38.51, *p* < 0.000001, $\eta_{{\text{p}}}^{2}$ = 0.28 (Fig. 1). Post-hoc comparisons reported a significant difference in accuracy between objects presented as PMs compared to those presented as PGs (*p* < 0.0001) or WD (*p* < 0.000001) and between objects presented as PGs compared to those presented as WD (*p* < 0.00003).

Since both false memories and inversions were scarce, only a descriptive analysis is provided here. Thirty-six of the 68 participants (53%) produced no false memories at all, 19 produced only one (28%) and 9 participants produced two (13%). With respect to format, 28 false memories were found in the space intended for PG (55% of false memories), 17 in that for PM (33%) and 6 in the space for WDs (12%). In line with previous studies (e.g., Helbig et al. 2006, 2010; Myung et al. 2006) in all but two cases, the reported object was semantically or functionally linked to at least one of the presented stimuli (*reported*/presented: e.g., *spoon*/knife; *screwdriver*/hammer; *lock*/key; *guitar*/drums). False memories were not specifically induced by one presentation format: for example, an erroneous report for a ‘guitar’ was found in participants who had viewed a photograph of ‘drums’ as well as in those who had seen the word ‘drums’, or the actor pretending to play the instrument. Of the 23 inversions, 11 referred to objects presented as WDs (48% of inversions), which were mainly reported as PGs. Seven inversions involved objects presented as PG (30%), which were mainly reported as PMs, and only 5 inversions involved items presented as PMs (22%), which were mainly reported as WDs.

### Recognition

On average participants correctly identified as old 26 of the 36 presented objects (*M* = 26.03, SD = 3.43) and correctly identified as new approx. 9 of the 12 distractors (*M* = 8.67, SD = 2.70) i.e., participants were over 70% accurate. In total there were only 103 false alarms and 104 source inversions (out of a total of 1456 responses, i.e., 7%). In fact, 86% of the responses provided were correct identifications or correct rejections. Cases in which participants did not respond (2.3%) or commented ‘not sure’, ‘don’t know’ (0.2%) were rare.

**Fig. 2:**
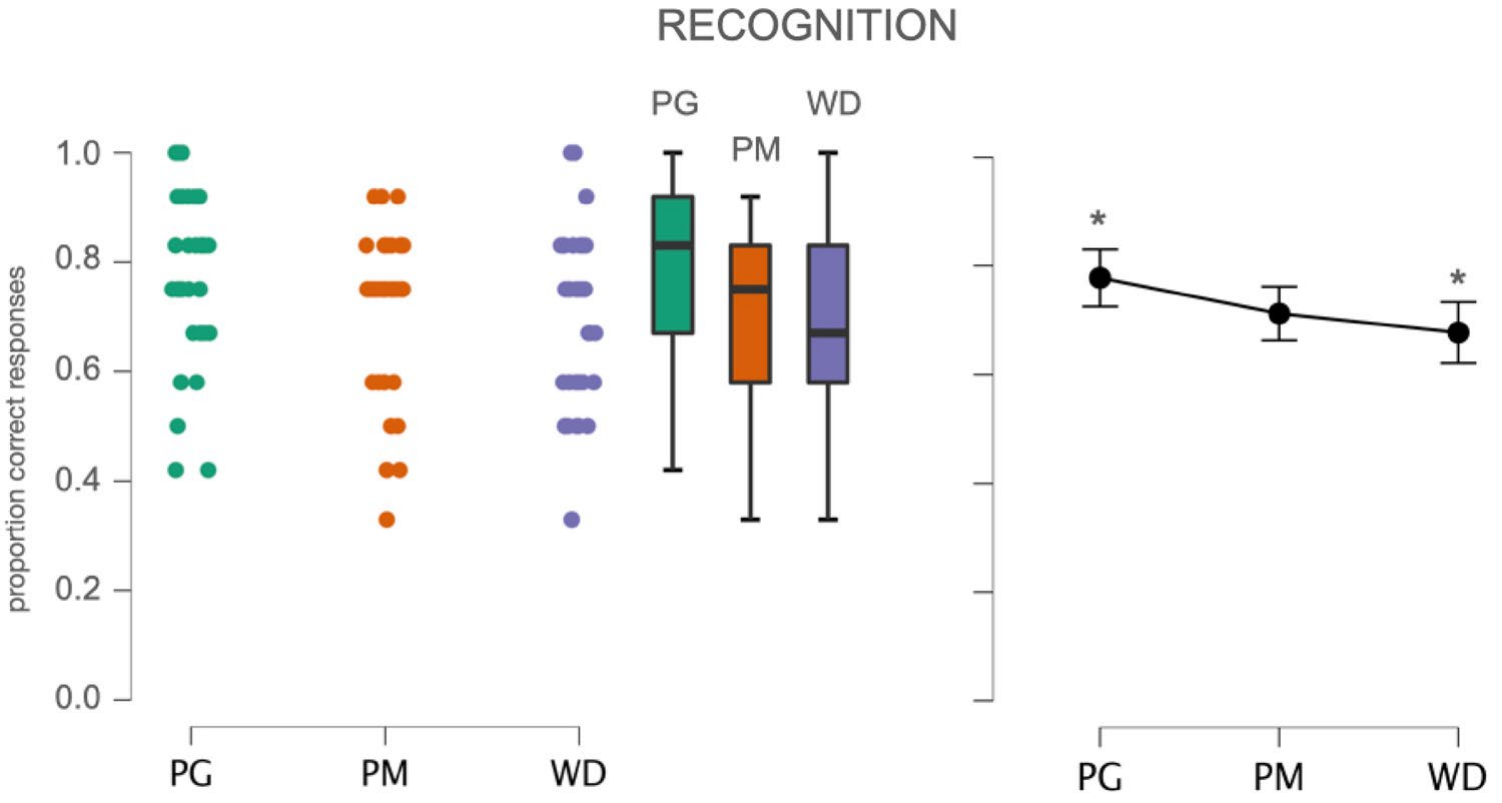
Accuracy for participants in the Recognition group (*N* = 36). Left: Jitter plots reporting all data points. Centre: Boxplot reporting median values (horizontal lines), interquartile range (top and bottom edges of the boxes). Whiskers extend to the most extreme data points. Right: Mean values (dot marker) and SEM. (whiskers). Asterisks highlight the significant post-hoc comparison: PG vs. WD, *t* = 2.67, *p* < .03. The remaining differences were not significant (PG vs. PM, *t* = − 2.14, *p* = 0.10; PM vs. WD, *t* = 0.53, *p* = 1). Abbreviations as for Fig. 1

Hit rate, expressed as the proportion of correct answers out of the total number of presented items, was as higher for objects previously presented as PGs (*M* = 0.78, SD = 0.16) and PMs (*M* = 0.71, SD = 0.15) than for objects that had appeared as WDs (*M* = 0.68, SD = 0.17). A one-way ANOVA on hit rates with presentation format (PG, PM, WD) as within-subjects factor showed a significant main effect of the former, *F*(2,105) = 4.01, *p* = 0.021, $\eta_{{\text{p}}}^{2}$ = 0.071 (Fig. 2). Post-hoc comparisons confirmed that significantly more items were correctly recognized when objects had appeared as PGs compared to when they had been presented as WDs (*p* = 0.026). The remaining comparisons were not significant (*p* > 0.05).

As mentioned above, false alarms and inversions were generally infrequent. The majority of the 103 false alarms was found among WDs (48%) and PGs (39%), while only 14% of novel objects erroneously labelled as old was assumed to have been presented as PM. The 104 source inversions were evenly distributed among items previously presented as WDs (33%), PGs (30%) and PMs (37%). In half of the cases (53%) the misrecognized object was judged to have been presented as WD. In 37% of cases, it was assumed to have been presented as PG, and only in 11% of cases as PM.

### Pantomimes

**Fig. 3:**
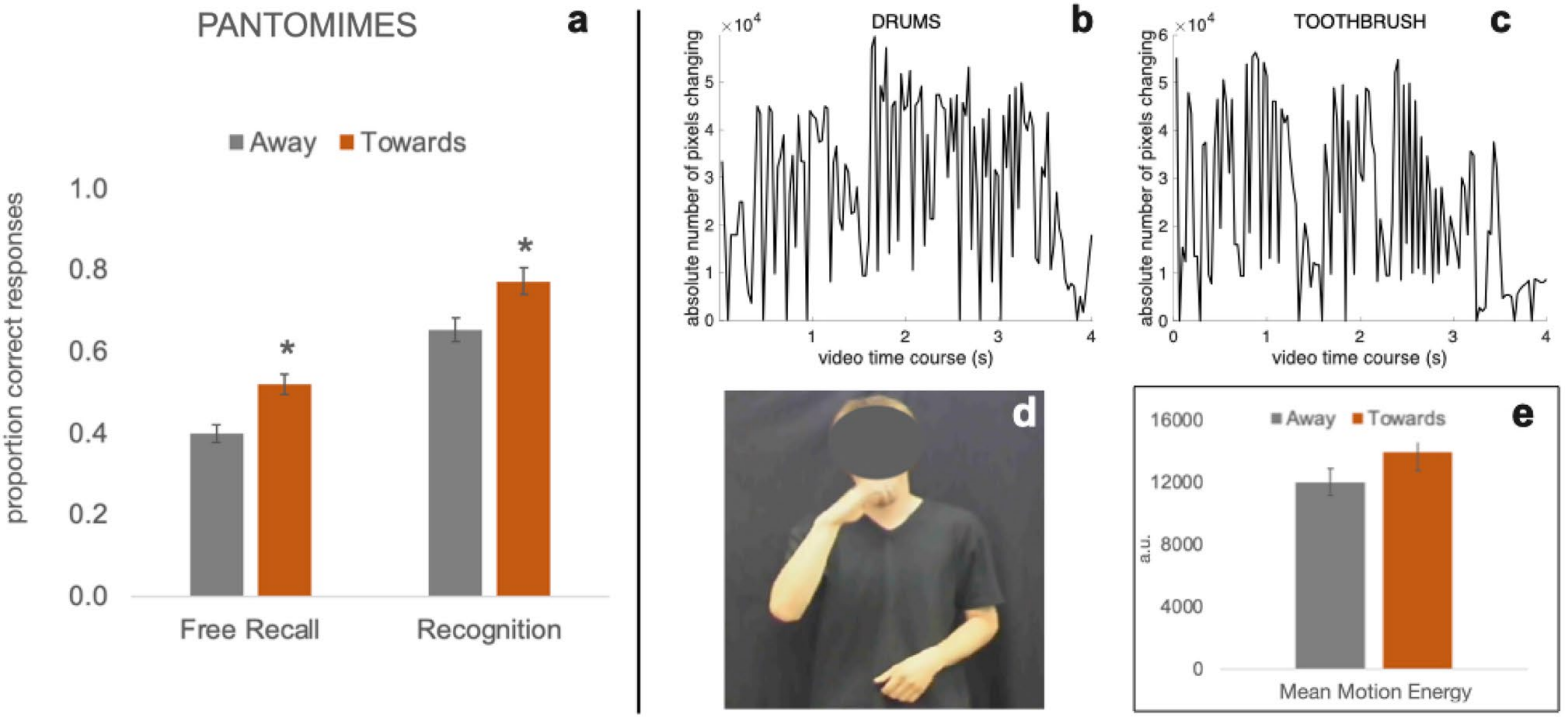
Results relative to objects presented as pantomimes. **a** Proportion of correct responses collected from participants in the Free Recall and Recognition groups split according to action direction (away/towards the body). Data refer to objects presented as pantomimes only. Grey bars: movement directed away from the actor’s body or towards another object (e.g., key, stones, drums). Red bars: movement directed towards the actor’s body or on his body (e.g., toothbrush, comb, hat). **b** Average level of motion energy contained in a video describing use of an object affording an action directed away from the body (drums). **c** Average level of motion energy contained in a video describing use of an object affording an action directed towards the actor’s body (toothbrush). **d** screenshot extracted from one of the videos (toothbrush). The actor’s face was visible to the participants during the experiment. **e** Mean motion energy (in arbitrary units, a.u.) for videos describing actions directed away and towards the actor’s body. Whiskers report SEM. Asterisks highlight the significant post-hoc comparisons

Following the lead of some participants’ comments, for items presented as PMs we inspected the characteristics of the actions afforded, in order to gain more insight into the advantage found in the Free Recall group. In the pantomimes viewed by all participants, 12 objects involved an action performed on or towards the actor’s body, (T) (binoculars, bottle, buttons, cigarette, comb, hat, glove, nail scissors, napkin, spectacles, telephone, toothbrush) and 12 objects involved an action directed away from his body or towards another object, (A) (drums, eraser, highlighter-pen, jar, knife, light bulb, needle, newspaper, playing cards, stones, tennis racket, wood saw). A t-test comparing hit rate for these two types of objects showed that participants freely recalled significantly more items if the objects afforded an action directed towards (*M* = 0.52, SD = 0.20) than away from the body (*M* = 0.40, SD = 0.18, *t* = − 3.81, *df* = 67, *p* < 0.0004, Cohen’s *d* =− 0.462) (Fig. 3, left section). The same difference emerged in the Recognition group: a significantly larger number of objects were correctly recognized if the action afforded was directed towards (*M* = 0.77, SD = 0.19) rather than away from the body (*M* = 0.65, SD = 0.17, *t* =− 2.72, *df* = 35, *p* = 0.01, Cohen’s *d* =− 0.454) (Fig. 3, panel a).

As we are dealing with dynamic stimuli, it could be that the difference described above reflects low-level aspects of the pantomime videos, such as presence of videos representing faster or broader movements, which could be more salient for the observer and therefore easier to remember. To rule out this possibility, we run a second analysis comparing the average level of motion energy contained in the videos of both categories. To do so, we processed each video with the Motion Energy Analysis tool (https://psync.ch/, Ramseyer 2020), an application that quantifies movement dynamics based on the color changes of pixels between consecutive frames. This frame-differencing program thus calculates the amount of change within a video and provides time-series of this quantification (see Fig. 3b and c for two examples). We computed the mean motion energy (MME) for each video (in arbitrary units, a.u.) by averaging the motion energy value calculated for each frame. We then compared the MME of videos describing actions directed away vs. towards the body by means of an unpaired t-test. It emerged that the mean MME for the two groups of videos did not significantly differ (A, *M* = 11,995, SD = 7089, *N* = 12; T, *M* = 13,952, SD = 9886, *N* = 12; (*t* =− 0.56, *df* = 19.95, *p* = 0.58, Fig. 3e).

*Summing up,* participants in both the Free Recall and the Recognition group correctly reported more objects previously presented as PGs than objects presented as WDs. In addition, participants in the Free Recall group remembered significantly more items previously presented as PMs than items that had appeared as PGs and WDs. For objects presented as PMs, participants in both groups showed better memory if the action afforded had been directed towards rather than away from the actor’s body (or towards another object). Average level of motion energy contained in the videos did not differ between the two object categories, ruling out an involvement of low-level, non-specific features of the dynamic stimuli.

## Discussion

We tested whether covert motor activity elicited by manipulable objects affects verbal memory. To this aim, we compared accuracy in remembering series of objects presented as verbal labels (the word describing the object, WD), visual images (color photographs, PG) or actions (pantomimes of object use, PM) and collected measures of Free Recall and Recognition memory in separate groups of participants. Two main findings emerged: (i) objects that had been presented as PGs or PMs were generally better remembered than objects presented as WDs; (ii) in the case of objects presented as PMs, the direction of the action afforded—either towards or away from the actor’s body—additionally influenced source memory.

The first result is in line with two phenomena described in the memory literature, namely the Picture Superiority Effect (PSE, Shepard 1967; Paivio and Csapo 1973) and the Dynamic Superiority Effect (DSE; Matthews et al. 2007; Buratto et al. 2009). The PSE is a robust finding that has been reported in both Free Recall and Recognition tasks. It describes the memory advantage for images compared to words and is assumed to depend on the fact that pictures allow for an item to be represented in two independent codes, visual and verbal. This richer encoding would lead to the storage of two memory traces (dual-code theory, Paivio 1991) and increase likelihood of later retrieving these items from memory. On the other hand, the DSE describes the memory advantage for dynamic images compared to static ones. This phenomenon has been mainly reported in studies presenting clips extracted from unfamiliar films depicting a variety of scenes including people, animals, vehicles, and natural views (Matthews et al. 2007; Buratto et al. 2009). It appears that when participants watch a moving clip, their memory is significantly more accurate compared to when they watch a sequence of snapshots extracted from the same movie. This effect persists for relatively long retention intervals (up to 1 week, Matthews et al. 2007) and has been linked to the larger amount of information contained in movies compared to static pictures, which may facilitate recall and/or keep participants more engaged, drawing their attention towards the study material (Buratto et al. 2009). In addition, given that fluid motion is a highly ecological stimulus, it has been suggested that motion may—by itself—improve recognition memory. In fact, “objects may change position […] but do not instantaneously disappear from one position to appear in another” (Matthews et al. 2007). Fluid progression of object motion is such a familiar—and predictable phenomenon that individuals tend to remember the endpoint of a viewed trajectory as if it were displaced further forward in the direction of the movement rather than at the correct location (representational momentum, Freyd and Finke 1984). This behavior suggests a bias in directing attention and fixation towards the moving target in an anticipatory fashion (DeLucia and Maldia 2006)—a pattern that supports the construction of rich memory traces where objects are anchored to spatiotemporal locations in a map (Matthews et al. 2007).

In line with reports on the PSE, an advantage for objects presented as PGs compared to WDs was observed here in both the Free Recall and the Recognition group, confirming that this effect can be detected also in relatively specific and homogeneous sets of stimuli (cf. also Job et al. 1992). In addition, participants in the Free Recall group remembered significantly more items previously presented as PMs than items that had appeared as PGs or WDs, further indicating a role for dynamic stimuli over static ones. Naturally, this advantage could reflect a phenomenon akin to the DSE. Pantomimes—i.e., the stimuli used here to convey a description of the objects via motor code—contain fluid motion (Matthews et al. 2007) and—as stimuli—are likely to be more engaging than photographs and words, both in consequence of the abundance of information they convey (Buratto et al. 2009) and of the need to understand what the actor is doing. As previously hypothesized for the DSE, the same factors could have boosted memory for the objects presented here as PMs. However, it could also be that the action-related component typical of pantomimes provided an additional motor code, which in turn made recall of these items more accurate compared to other formats. It has been observed that images (and by the same token, words) may be capable to elicit affordances but lack *actability*, i.e., the possibility to interact with the object they represent (Snow and Culham 2021). Pantomimes, on the other hand, are actions and may be more likely to provide a motor signature to the overall experience. Several data indicate that the advantage derived from encoding items in more than one code is not limited to the visual modality: effects comparable to the PSE have been previously described for sounds (Crutcher and Beer 2011) and odors (Lyman and McDaniel 1990). Besides, it has been reported that when individuals can associate motor codes to a piece of verbal information (such as actively performing the action described in a sentence instead of simply listening to it), their memory for the verbal material significantly improves (Enactment Effect, EE; Engelkamp 1998; Feyereisen 2006). Neuroimaging studies investigating this effect reported that recall processes for items encoded via enactment further engage the sensorimotor system suggesting that memory relies on activating and/or re-activating the additional motor information (Nilsson et al. 2000; Nyberg et al. 2001; Russ et al. 2003). A similar phenomenon might have occurred here, covert motor activity providing an individual—accessory—motor code. Indeed, watching an individual performing an action is a compelling experience, which goes beyond the non-specific effects produced by observing dynamic stimuli. As anticipated in the Introduction—the sight of an action can induce in the observer multiple motor responses according to context (cf. Orban et al. 2021b): imitation (Heyes 2011; Cracco et al. 2018) is an option, as is preparing a complementary action, such as receiving an offered object or cooperating in using it. Phenomena of motor resonance are assumed to largely map onto the mirror neuron system of the observer (MNS), a brain network activated by execution and observation of the same motor act (Bonini et al. 2022). Accordingly, in the case of objects presented as PMs, the covert motor activity evoked by the stimuli could have enriched the memory trace in much the same way as overt and imagined actions do (Denis et al. 1991). In other words, in the case of objects presented as PMs, stimuli may have induced a stronger first-person experience in the participants, which in turn could have boosted memory for the objects described by the pantomimes.

It should be acknowledged that differently from the PSE, which was detected in all participants, the advantage for objects presented as PMs failed to emerge in the Recognition group. This could depend on the smaller number of participants involved and/or the relatively lower complexity of the task, which somewhat leveled performance. However, it could also be that the structure of the task failed to induce the same strong, individual experience as Free Recall. In fact, to access stored information, participants in the Recognition group were not obliged to generate their own recall cues since they were offered a list of items to evaluate as old or new. This process may have reduced first-person involvement during recall, failing to re-activate the motor codes produced by pantomimes during the encoding phase. Our second finding seems to partly support this claim, as discussed below.

In both the Free Recall and the Recognition group, accuracy in remembering objects presented as PMs increased if the action afforded was directed towards the actor’s body. This peculiar finding is unlikely to depend on some low-level, non-specific feature contained in the dynamic stimuli, as average level of motion energy contained in the videos did not differ between the two object categories. More likely, this advantage could be due to the characteristics of the involved stimuli and of the space where actions take place. Self-directed movements are performed within a highly sensitive region of space (peri-personal space, e.g., Rizzolatti et al. 1997). The relation between objects, body parts and the portion of space they share while interacting is an extremely peculiar one. An ample literature shows that objects can be incorporated in body representations, eventually producing consistent remapping of the peri-personal space (for a review, see Martel et al. 2016). In the present case, such a strict connection may have acted favorably on recall of objects involved in self-directed actions, possibly by inducing stronger first-person engagement on the part of the participants. Besides, there are several reasons why objects involved in actions directed towards the body might be generally considered as salient. Data from the developmental literature suggest that movements directed towards the body are more familiar, require better visuomotor control (Njiokiktjien et al. 2000) and a higher degree of planning (Claxton et al. 2009) compared to actions directed towards the external world. In addition, evidence from kinematics studies indicates that actions directed towards the body can be selectively affected by the emotional valence of the moved object (e.g., mobile phone vs. artificial spider; Esteves et al. 2016). In line with these data, observations from neurophysiology further suggest that signals evoked by objects involved in self-directed actions may be dealt with by entirely different neural substrates from those involved in manipulative actions, in accordance with the diverse type of action they may afford (Orban et al. 2021a). Stimuli touching the skin (Cooke and Graziano 2003) for example, and/or the sight of objects looming towards the body (Orban et al. 2021b) are likely to activate defensive or avoidance responses. From an ecological perspective, all these characteristics are consistent with the fact that the risks of negative consequences are more relevant to the self when objects are used in actions directed towards—rather than away from—the body (Claxton et al. 2009; Esteves et al. 2016). Action representations may thus be more substantial and relevant to objects affording the former type of actions. On these bases, it can be assumed that watching pantomimes describing objects that are used on one’s body (such as a comb or a toothbrush) produced stronger first-person engagement on the part of the observer and made the associated motor experience more ‘personal’ and possibly relevant. Stated differently, observation of pantomimes of actions directed towards the actor’s body may have provided a stronger drive towards assuming a first-person perspective in the observers, making covert motor activity more prominent. Consequently, memory for objects affording this type of movement could have been enriched by the additional episodic trace provided by the covert action elicited by the pantomime. As a matter of fact, if only objects affording actions directed towards the actor’s body are considered, memory for items presented as PMs was superior to that for objects presented as PGs also in the Recognition group (PM, *M* = 0.77, SD = 0.19; PG, *M* = 0.65, SD = 0.27). Of course, in this case we can only speculate that the actions evoked by the photographs matched on those presented in the videos, but this is not unlikely given that objects requiring an action towards the body have been found to automatically activate the direction of object use in a verbal categorization task (Scotto di Tella et al. 2021). Albeit caution is needed, this advantage speaks in favor of a direct link between stronger drive towards assuming a first-person stance and increased memory accuracy.

Two further observations deserve attention. First, participants in the Free Recall group retained better memory for objects presented as PMs, i.e., for objects that had not been physically there. Remembering something that has not been really experienced is not surprising per se. Empirical demonstrations and models exist in support of the ‘reconstructive’ nature of episodic memory (Schacter and Addis 2007). Indeed, a coherent memory of an event can be created by putting together elements of past experiences with information derived from inferential processing (Binte Mohd Ikhsan et al. 2020). Here an analogous mechanism could have supported reconstruction of the objects presented as pantomimes, causing a deeper, more extensive, encoding process that subsequently led to the more stable memory traces. Alternatively, the advantage of a ‘suggested’ target over truly experienced ones could depend on the fact that pantomimes provided a context into which framing the object. Scenes facilitate binding, possibly by providing a spatial scaffold for association and integration across items (Robin and Olsen 2019). Likewise, the observed action could have created a setting encompassing the target and the semantic content related to it, adding ecological salience to the event coded in episodic memory, reinforcing its trace.

Second, in the current study actions were either implied or witnessed—but never performed—by participants. Previous data provide mixed evidence with respect to whether incidental motor information is stored in memory and contributes to further characterizing experiences (Zeelenberg and Pecher 2016). In right-handers, memory of where a series of cups must be placed was reported to improve if their handles were oriented to the right (Apel et al. 2012), suggesting that the effects of motor affordances translate into memory. Similarly, memory for objects’ pictures was interfered by grasping movements presented prior to recall (Downing-Doucet and Guerard 2014), although a motor-interference task performed between two successive stimuli (Pecher 2013) (or concurrently; Quak et al. 2014) had no such effect. Nor were compatibility effects between target and response maintained in memory tasks (Canits et al. 2018). In fact, reliability of the well-known Action-sentence Compatibility Effect (Glenberg and Kaschak 2002) has recently been reviewed (Morey et al. 2022), questioning the role of motor activity in verbal tasks. Notwithstanding the many methodological differences, we think that these discordant findings could be reconciled on account of the unequal resonance produced by the tasks within the motor system. If motor affordances are highlighted, predicting some form of action simulation, as in Apel et al. (2012), Guerard and Lagace (2014) or Lagace and Guerard (2015), motor-dependent effects can extend to memory, whereas if emphasis on the movement is less specific (Pecher 2013) these effects are reduced. Likewise, here hit rates were significantly higher for objects presented in a format that directly evoked action representations (PMs) compared to a format that indirectly called for the contribution of the sensorimotor system (WDs).

The present study is preliminary and suffers from some limitations, the most notable being the lack of direct information on sub-threshold motor activity during the encoding phase. Future studies should be run to investigate this parameter with appropriate methodologies. In addition, all representations—including pictorial ones—were 2D images displayed well beyond the limits of the participants’ reaching space. While this was purposefully done to mimic accidental encounters with objects that do not involve actual interactions (as happens when casually looking at a billboard while driving), this peculiar setup could have played on objects’ affordances. As previously mentioned, so-called variable affordances, i.e., affordances directly related to the to-be-performed actions (Borghi and Riggio 2009, 2015) appear to be context-dependent and their effectiveness is reduced if stimuli are not immediately within reach (Costantini et al. 2010, 2011). It is thus possible that—had 3D images been presented within arm reach—accuracy for items presented as PGs could have improved. Despite these limits, we believe our findings suggest the possibility that the early phases of motor activity—such as intention-to-act and/or planning—could be differently involved according to how objects are experienced, and, ecologically, according to the direction of the actions. This ‘motor tag’ would better characterize the single experience, providing information that supports accurate source memory.

One last remark may be relevant here. Recent theories of cognition consider concepts referring to physical entities (such as objects) as grounded within the systems that support perception and action, of which they would share—at least in part—the neural bases (Lakoff and Johnson 1980; Semin and Smith 2008; Barsalou 2008, 2010; Wilson 2002; Borghi and Pecher 2011). Within this framework the differential effect on memory reported here for objects presented as WDs, PGs and PMs could be viewed as depending on the different levels of cognitive embodiment produced by the three formats and the degree of re-enactment they are likely to evoke. If one assumes that conceptual knowledge about objects is grounded in the neural networks supporting the sensorimotor experience that people acquire of them (e.g., Buccino et al. 2016; Aflalo et al. 2020), it follows that this knowledge can be affected by variability in these experiences, as also highlighted by the role played by context (e.g., Costantini et al. 2010; Wokke et al. 2016; Buccino et al. 2016; Orban et al. 2021a, b; for discussions on this topic, see also Borghi and Binkofski 2014; Barsalou 2020; Mazzuca et al. 2021). Here, the experience of photographs would have been more definite than that for words that do not describe a precise item (in terms of color or size) but rather refer to a category of objects (e.g., balls, toothbrushes), and this difference could have affected the degree of re-enactment involved by the two object formats. In a similar way, the increased accuracy recorded for objects described by actions could be warranted by the large amount of action information conveyed by this type of stimuli that would allow for richer re-enactment. Likewise, the advantage found for objects involved in pantomimes directed towards the body could depend on the strong emotional value associated to stimuli located in proximity to the self. In fact, these stimuli are more likely to activate defensive or avoidance responses (Cooke and Graziano 2003; Orban et al. 2021a; Claxton et al. 2009; Esteves et al. 2016) and to present with stronger internal salience, considering the many autonomic and visceral responses associated to emotional experiences (e.g., interoception, Connell et al. 2018). Compared to pantomimes directed away from the body, these stimuli could thus have caused deeper embodiment and re-enactment, a phenomenon that could further justify the memory accuracy participants showed for the associated (though invisible) objects. In this view, the format-based modulation in memory accuracy reported here supports the hypothesis that motor information may, at different levels, partake in concept representation.

## Data Availability

The dataset relative to the current study is not publicly available due to privacy reasons as it contains sensitive information on the participants. It is available from the corresponding author on reasonable request and under confidentiality agreement.

## Appendix 1: List of the objects used in the study

1. *Presented items* Participants were presented with 36 objects that could appear as a verbal label (WD), a color photograph (PG) or a pantomime of the object’s use (PM). In the latter case, the real object was never visible but could easily be recognized from the action performed by the actor. The action performed was always highly paradigmatic and clearly referred to the object’s most characteristic use (e.g., toothbrush = brushing one’s teeth; paintbrush = painting, etc.). Thus, albeit absent, the object was unmistakably recognizable (see Daprati et al. 2005 for details on these stimuli).

The 36 objects were divided in three 12-items lists (A, B, C). Across lists, object names did not differ in terms of word length (list A, *M* = 7.2, range 3–13; list B, *M* = 8.3, range 5–10; list C, *M* = 7.9 range 6–10, *t* tests all *p* > 0.4) and word frequency (list A, *M* = 56.4, range 1–280; list B, *M* = 51.9, range 4–234; list C, *M* = 50.3 range 1–212, t-tests all *p* > 0.8), as obtained from the Italian database CoLFIS (Laudanna et al. 1995). Objects from one list were presented as either PG, PM or WD to separate groups of participants, so that the same item eventually appeared in all formats albeit to different participants (i.e., object 1 was presented as WD to participant 1, as PG to participant 7 etc.).

The presented objects are listed below.

Belt, binoculars, bottle, buttons, cigarette, comb, drinking glass, drums, eraser, glass wiper, glove, hat, highlighter pen, jar, key, knife, lightbulb, marbles, nail scissors, napkin, needle, newspaper, paintbrush, pistol, playing cards, rolling pin, spectacles, spinning top, stapler, stones, sweater, telephone, tennis racket, tooth-brush, tweezers, wood saw.

2. *Novel items* For the Recognition Task, an additional 12 objects were included in the recognition grid and acted as distractors. These items are listed below.

Ball, dice, hammer, ice-cream, iron, lighter, pencil-sharpener, piano, scarf, soap, spoon, wrist watch.

Names of the objects included in this list did not differ from those of the items included in the previous three lists in terms of word frequency (list *N*, *M* = 55.2, range 4–188, all *p* > 0.8) and word length (*M* = 7.4, range 4–11, all *p* > 0.2).

## Footnotes

1 Each object is behavior transformed into a thing and then retransformed into behavior.
